# Rapid Emergence of Cefiderocol Resistance Associated with Mutation of *EnvZ* Gene in a VIM-Producing ST307 *Klebsiella pneumoniae* Strain

**DOI:** 10.3390/antibiotics14090893

**Published:** 2025-09-04

**Authors:** Simone Ambretti, Benedetta Secci, Raul Cetatean, Milo Gatti, Pierluigi Viale, Federico Pea, Claudio Foschi

**Affiliations:** 1 Microbiology Unit, IRCCS Azienda Ospedaliero-Universitaria of Bologna, 40138 Bologna, Italy; simone.ambretti@aosp.bo.it (S.A.); benedetta.secci@aosp.bo.it (B.S.); 2 Department of Medical and Surgical Sciences, Alma Mater Studiorum-University of Bologna, 40138 Bologna, Italy; milo.gatti2@unibo.it (M.G.); pierluigi.viale@unibo.it (P.V.); federico.pea@unibo.it (F.P.); 3 Department of Public Health, Experimental and Forensic Medicine, University of Pavia, 27100 Pavia, Italy; raul.cetatean2@unibo.it; 4 Clinical Pharmacology Unit, IRCCS Azienda Ospedaliero-Universitaria of Bologna, 40138 Bologna, Italy; 5 Infectious Diseases Unit, Department for Integrated Infectious Risk Management, IRCCS Azienda Ospedaliero-Universitaria of Bologna, 40138 Bologna, Italy

**Keywords:** *Klebsiella pneumoniae*, resistance, cefiderocol, whole-genome sequencing (WGS)

## Abstract

Infections caused by carbapenem-resistant *Enterobacterales* (CRE), particularly those harboring metallo-β-lactamases (MBLs) such as VIM, constitute a significant public health threat due to the paucity of effective therapeutic options. Cefiderocol (CFD), a novel siderophore-conjugated cephalosporin, exhibits potent activity against CRE by exploiting bacterial iron uptake systems. Nevertheless, the emergence of CFD resistance has been recently documented. This study aimed to characterize the development of CFD resistance in a VIM-producing *Klebsiella pneumoniae* isolate during antimicrobial treatment. Antimicrobial susceptibility was assessed by broth microdilution using iron-depleted medium according to EUCAST guidelines. Whole-genome sequencing and comparative genomic analyses focused on mutations in genes related to iron transport and CFD resistance, using Illumina MiSeq. Initial isolates (RS, BA1) were susceptible to CFD (MIC 2 mg/L), whereas the isolate recovered after 9 days of CFD therapy (BA2) was resistant (MIC 8 mg/L). In conclusion, this study illustrates for the first time the rapid emergence of CFD resistance in a VIM-producing ST307 *K. pneumoniae* isolate linked to a missense variant in *envZ* gene, arising after a 9-day CFD treatment.

## 1. Introduction

Infections caused by carbapenem-resistant *Enterobacterales* (CRE) are increasing globally and represent a major public health threat [1]. Among the mechanisms driving carbapenem resistance, the production of carbapenemases enzymes capable of hydrolyzing carbapenems and other β-lactams is the most clinically significant. These enzymes are classified into different molecular classes, including serine carbapenemases (e.g., KPC, OXA-48) and metallo-β-lactamases (MBLs) such as VIM, IMP, and NDM [2]. Among the different enzymes, MBLs, such as VIM, are still highly difficult to treat considering that most of the new generation inhibitors (avibactam, vaborbactam, relebactam) are not active against this class of carbapenemases [1]. These enzymes are often encoded on mobile genetic elements, facilitating their dissemination across species and clinical settings, and are commonly associated with high-risk clones such as *Klebsiella pneumoniae* ST307, which has been implicated in numerous nosocomial outbreaks worldwide. In this context, the search for effective treatment alternatives has become a global priority, and novel agents with broad-spectrum activity against CRE are being increasingly investigated. The limited armamentarium against MBL-producing CRE has spurred the development of novel antimicrobial agents [3,4]. A novel siderophore cephalosporin, Cefiderocol has recently gained increasing attention for its in vitro activity against CRE, including MBL producers [5]. This siderophore molecule, utilizing natural iron transportation systems, is actively transported across the outer membrane of Gram-negative bacteria, therefore overcoming resistance mediated by porin loss or efflux pumps [6]. However, despite the promising activity against most β-lactam resistance mechanisms, alarming proportions of CFD resistance have been reported in different bacterial species in some recent cohorts [6].

Here, we describe a case of a VIM-producing *Klebsiella pneumoniae* strain developing CFD resistance after treatment. In a 51-year-old male, admitted for orthotopic liver transplant at IRCCS Azienda Ospedaliero-Universitaria of Bologna (Italy), a rectal colonization by a VIM-producing *K. pneumoniae* was documented during active surveillance for carbapenem-producing *Enterobacterales*. During hospitalization, the patient needed two further liver transplants (day 7 and 45) and suffered from several bloodstream infections and ventilator-associated pneumonias (VAP) caused by a VIM-producing *K. pneumoniae*. The patient was treated with ceftazidime–avibactam plus aztreonam, until day 52 when treatment with CFD was started. Exposure was optimized by therapeutic drug monitoring (TDM)-guided approach, but a bronchoalveolar lavage performed on day 61 yielded a VIM-producing *K. pneumoniae* strain resistant to CFD. Further details of the case are reported in Gatti et al., 2025 [7].

We included and analyzed three different *K. pneumoniae* strains from this patient. The first strain was isolated from a rectal swab (RS) performed at the beginning of hospitalization and the two subsequent strains were recovered from lower respiratory tract samples at days 10 and 61 (BA1 and BA2, respectively). This study illustrates for the first time the rapid emergence of CFD resistance in a VIM-producing ST307 *K. pneumoniae* isolate linked to a missense variant in *envZ* gene, arising after a 9-day CFD treatment.

## 2. Results

### 2.1. Phenotypic Analysis

The clinical strains (RS, BA1 and BA2) tested positive for the production of a VIM carbapenemase via a rapid phenotypic immunochromatographic assay. This finding was corroborated by the detection of the *bla_VIM_* gene by a commercial nucleic acid amplification test (Xpert Carba-R; Cepheid, Sunnyvale, CA, USA).

Synergy with ethylenediaminetetraacetic acid confirmed a class B carbapenemase, whereas a positive carbapenem inactivation test highlighted the ability of the carbapenemase to hydrolyze meropenem.

As shown in detail in Table 1, the three strains of *K. pneumoniae* (RS, BA1, BA2) are resistant to Ampicillin, Amoxicillin/clavulanic acid, Ceftazidime + Avibactam, Ceftazidime, Ceftolozane + Tazobactam, Ciprofloxacin, Cefotaxime, Ertapenem, Meropenem, Meropenem + Vaborbactam, Sulfamethoxazole/trimethoprim, Piperacillin + Tazobactam, Aztreonam, Cefepime, Eravacycline, Imipenem, Imipenem + Relebactam, and Tobramycin. Conversely, susceptibility was noticed for Amikacin and Gentamicin in all three isolates.

To evaluate potential fitness differences, we performed an analysis of bacterial growth curves for BA1 and BA2 isolates under iron-rich and iron-depleted conditions. Both isolates displayed enhanced growth in the presence of iron compared to iron-limited medium. Additionally, BA1 exhibited an increased growth compared to BA2 in the iron-rich medium (Supplementary Figure S1).

The first two strains (i.e., RS and BA1) were susceptible to CFD with a MIC of 2 mg/L, whereas the third strain (i.e., BA2), obtained after a 9-day-treatment with CFD, showed a MIC of 8 mg/L, being categorized as resistant following EUCAST guidelines (https://www.eucast.org/clinical_breakpoints, accessed on 20 August 2025).

To evaluate the possible presence of heteroresistance in BA2 isolate, we performed both MIC Test Strip and disk diffusion assays for CFD, carefully examining the inhibition zones for the appearance of inner colonies. Even though additional approaches are needed for the assessment of heteroresistance (e.g., population analysis profile), the absence of inner colonies in our assays presumably suggests that the isolate does not exhibit a heteroresistant phenotype.

### 2.2. Genomic Characterization of the Isolates

Whole-genome sequencing was performed for the three *Klebsiella pneumoniae* isolate (RS, BA1, BA2). The assembled genome sizes were 5,990,110 bp, 5,990,048 bp, and 5,955,006 bp for RS, BA1, and BA2, respectively. De novo assembly resulted in 198, 164, and 187 scaffolds with corresponding N50 values of 110,098 bp, 179,138 bp, and 129,094 bp for RS, BA1, and BA2. Scaffolds shorter than 200 bp were excluded from the assembly metrics. The GC content was 56.62% for isolates RS and BA1, and 56.63% for BA2. Genome annotation identified a total of 5717 genes in RS, including 5638 protein-coding sequences (CDSs); 5712 genes (5629 CDSs) in BA1; and 5669 genes in BA2, of which 5585 were CDSs.

All three isolates belonged to the sequence type 307 (gapA(4), infB(1), mdh(2), pgi(52), phoE(1), rpoB(1), tonB(7)), and shared the same resistance genes, with five beta-lactamase genes among them: blaTEM-206, blaCTX-M-15, blaOXA-1, blaSHV-12, and blaVIM-1 (Table 2).

The analysis did not reveal the presence of any virulence factors for the examined isolates.

Capsular and O-antigen typing revealed that all strains possessed the polysaccharide KL102 capsular locus (99.6% identity) and a lipopolysaccharide O1/O2v2 locus similar to O2afg type (99% identity).

The plasmids identified with an identity >91.5% were as follows: Col(pHAD28) (KU674895), IncA (FJ705807), IncFIB(K) (JN233704), IncFIB(pNDM-Mar) (JN420336), IncFII(K) (CP000648), and RepB (CP061702.1).

### 2.3. Analysis of Iron Uptake and Transport Genes

To investigate the mechanism behind CFD resistance, the assembled genomes were compared against the *K. pneumoniae* genome 48-IT, focusing on genes involved in iron uptake and transport, associated with CFD resistance [6,8]. The isolate BA2 (the only exhibiting a phenotypic resistance to CFD) harbored a missense variant in the *envZ* gene (Ala231Val), which was not present in the other two isolates.

## 3. Discussion

For the first time in this study, a previously unreported mutation of the *envZ* gene linked to CFD resistance in a VIM-producing *K. pneumoniae* clinical isolate has been described.

The *envZ* gene encodes for the kinase of the EnvZ/OmpR, a two-component system involved in the regulation of outer membrane porins such as OmpC and OmpF; this system has also recently been shown to play a role in iron transport.

Gerken et al. [9] suggested that constitutive *envZ* gene has a crucial role activating the Feo- and OmpC-mediated iron uptake pathways, flooding the cytoplasm with available ferrous iron.

Mutations in siderophore receptors (piuA, cirA, fiuA) and other components (tonB, envZ) involved in iron uptake have been observed in various species such as *Acinetobacter baumannii*, *Pseudomonas aeruginosa*, *Enterobacter cloacae*, and *Klebsiella pneumoniae* [10]. Among them, *envZ* has been identified as a hotspot for mutations in in vitro CFD resistance studies. Indeed, Kriz et al. [10] defined *envZ* as the gene with the higher number of variants selected after CFD exposure. Several different mutations (L27P, R82C, I86S, V145G, T247I, T247P) were described as affecting CFD uptake and causing resistance. These findings suggest that mutations in *envZ* play a major role in CFD resistance.

Moreover, Findlay et al. [11] recently reported a new mutation in *envZ*, resulting in a Val147Gly change, associated with CFD resistance in a KPC-producing *K. pneumoniae* clinical isolate.

Yang et al. [12] further investigated the combined effect of carbapenemase production and expression of genes involved in iron uptake. The authors identified a missense mutation of *envZ* (T434G) in all sequenced strains resistant to CFD, confirming that missense alleles of this gene can negatively affect the expression of iron uptake genes.

At the moment of writing, the Ala231Val mutation has not been reported to be involved as a factor in CFD resistance. To further investigate whether the identified mutation is exclusive to our isolate BA2, *K. pneumoniae* genomes with sequence type 307 were downloaded from NCBI. The genomes were then annotated and the *envZ* sequences were extracted and aligned with our isolates. The multiple sequence alignment determined that the Ala231Val mutation is uniquely present in isolate BA2.

Interestingly, as previously suggested by other authors [11], we observed that the mutation in the *envZ* gene imposed a fitness cost on *K. pneumoniae* BA2 isolate (i.e., reduced growth compared to BA1 in iron-rich medium). Considering that iron is essential for bacterial cell function, the limited uptake could presumably compromise cell fitness.

We are fully aware of some limitations of the study. At first, although our findings indicate that isolates BA1 and BA2 are closely related, it is not possible to determine with complete certainty whether BA2 originated from BA1 or if the isolates were independently acquired by the patient. Moreover, other studies are needed for a more in-depth phenotypic and genotypic characterization of BA1 and BA2 isolates, including specific assays for comparing iron uptake capabilities and heteroresistant phenotypes.

In conclusion, alterations in *envZ* gene had been increasingly recognized as contributors to resistance to CFD in *Enterobacterales*. This study illustrates for the first time the rapid emergence of CFD resistance in a VIM-producing ST307 *K. pneumoniae* isolate presumptively linked to a missense variant in *envZ* gene, arising after a 9-day CFD treatment.

## 4. Materials and Methods

### 4.1. Bacterial Strains and Antimicrobial Susceptibility Testing

At the IRCCS Azienda Ospedaliero-Universitaria of Bologna (Italy), a 51-year-old male admitted for orthotopic liver transplantation was found to carry a rectal colonization by a VIM-producing *K. pneumoniae*, identified through active screening for carbapenemase-producing *Enterobacterales*. The same microorganism was also isolated from the lower respiratory tract.

Briefly, the first strain was isolated from a rectal swab (RS; deposited as CV4 BioSample SAMN46786795 at NCBI; https://www.ncbi.nlm.nih.gov/bioproject/); subsequent strains were recovered from lower respiratory tract samples at days 10 and 61 (BA1 and BA2; deposited as CV5 BioSample SAMN46862875 and CV2 BioSample SAMN46786351, respectively).

The three bacterial strains included in this study were identified at the species level by MALDI-TOF mass spectrometry (Bruker Daltonik GmbH, Bremen, Germany), whereas VIM production was confirmed by a multiplex immunochromatographic assay (NG-Test CARBA 5; NG Biotech, Guipry, France), and a commercial NAAT (Xpert Carba-R; Cepheid, Sunnyvale, CA, USA).

Additionally, we performed the carbapenem inactivation method (CIM) to confirm carbapenemase activity, as well as a synergy test (combination disk testing with meropenem ± various inhibitors) to identify the carbapenemase class, following the EUCAST guidelines (https://www.eucast.org/resistance_mechanisms, accessed on 20 August 2025). Antimicrobial susceptibility testing was conducted using the MicroScan WalkAway system. Susceptibility to Aztreonam, Cefepime, Colistin, Eravacycline, Imipenem, Imipenem/Relebactam, Tigecycline, and Tobramycin was assessed using the Sensititre™ EUMDRXXF plate (Thermo Fisher Scientific, Waltham, MA, USA). For Cefiderocol (CFD), susceptibility testing was performed via broth microdilution in an iron-depleted medium, following the EUCAST guidelines (https://www.eucast.org/fileadmin/src/media/PDFs/EUCAST_files/Guidance_documents/Cefiderocol_MIC_testing_EUCAST_guidance_document_January_2024.pdf, accessed on 20 August 2025).

To evaluate the possible presence of heteroresistance in BA2 isolate, we performed both MIC Test Strip and disk diffusion assays for CFD (Liofilchem, Teramo, Italy).

To evaluate phenotypic variations in BA1 and BA2 isolates under different conditions, microbial growth curves were performed for both isolates in iron-rich and iron-depleted media. Bacterial growth was monitored by measuring optical density over time (Supplementary Figure S1).

### 4.2. Whole-Genome Sequencing and Bioinformatic Analyses

Whole-genome sequencing and genomic data analysis were carried out as follows. Genomic DNA was extracted by DNeasyBlood&Tissue Kit (Qiagen, Hombrechtikon, Switzerland), whereas Illumina paired-end libraries were generated using DNA Prep Library Preparation Kit (Illumina, San Diego, CA, USA). Sequencing was performed with Illumina MiSeq platform.

Fastqc was used to check for the quality of the raw reads (https://github.com/s-andrews/FastQC, accessed on 10 November 2024). Trimming of the short reads was performed with trim_galore v.0.6.10 using automatic adapter detection (https://github.com/FelixKrueger/TrimGalore, accessed on 10 November 2024). A minimum read length of 50 bp and a Phred quality cutoff of 20 were applied. Fastqc was then re-run for the quality control of the trimmed reads to check also for the correct removal of the adapters. De novo genome assembly and annotation were performed using SPAdes version 4.0.0 and Prokka version 1.14.6 [13,14]. Spades was run using the isolate flag, as it is highly recommended for Illumina data. Prokka parameters were optimized for bacterial genomes. The kingdom Bacteria, genus *Klebsiella* and species *pneumoniae* flags were specified in the command line to ensure accurate functional predictions. The usegenus flag was included in order to access genus-specific BLAST databases (version 2.17.0) for improved annotation quality. Prokka was executed using multiple threads with the cpus option. In silico multi-locus sequence typing was determined using the mlst command line program against the PubMLST typing schemes (https://github.com/tseemann/mlst, accessed on 10 November 2024) [15]. Antimicrobial resistance genes were identified using the NCBI Antimicrobial Resistance Gene Finder software AMRFinderPlus (version 3.12.8) [16]. The tool was run with the annotation-format option, allowing the program to parse the output files from the Prokka. The organism and the plus flags were also used to obtain organism-specific results and to obtain virulence factors, stress-response genes, and other genes of interest present in the AMRFinderPlus database. PlasmidFinder software (version 2.1) was used to detect the presence of plasmids [17]. The analysis was automated using a Snakemake script [18]. Quality of the assemblies was assessed with Quast version 5.3.0. [19].

Capsular and O-antigen typing were performed using Kleborate version 3.1.2 by using the klebsiella_pneumo_complex__kaptive module available from the command line [20,21].

Variant calling was performed using Snippy version 4.6.0 (https://github.com/tseemann/snippy, accessed on 10 November 2024). In particular, the snippy-multi command was employed, allowing us to compare our isolates against the same reference. The assembled genomes were compared against the *K. pneumoniae* 48-IT (accession number: PRJNA295649) genome, and a custom python script was implemented to extract the genes involved in iron uptake and transport. The custom python script screens for mutations in genes associated with iron uptake and transport, which have been previously implicated in Cefiderocol resistance based on a literature review.

*Klebsiella pneumoniae* genomes with sequence type 307 were downloaded from NCBI (https://www.ncbi.nlm.nih.gov/datasets/genome/, accessed on 3 June 2025) and annotated using Prokka as previously described. *EnvZ* gene sequences were extracted and then aligned together with the sequences of our isolate EnvZ in order to establish if the mutation is exclusive to isolate BA2. The alignment was performed with mafft version 7.526 using the auto flag [22]. 

## Figures and Tables

**Table 1:** Phenotypic characteristics of clinical isolates included in this study.

	MIC Value (mg/L)		
Antimicrobial	RS	BA1	BA2
Amikacin	≤8	≤8	≤8
Ampicillin	>8	>8	>8
Amoxicillin/clavulanic acid	>32	>32	>32
Ceftazidime + avibactam	>8	>8	>8
Ceftazidime	>32	>32	>32
Ceftolozane + Tazobactam	>4	>4	>4
Ciprofloxacin	>1	>1	>1
Cefotaxime	>32	>32	>32
Ertapenem	>1	>1	>1
Gentamicin	≤2	≤2	≤2
Meropenem	32	32	32
Meropenem + Vaborbactam	>16	>16	>16
Sulfamethoxazole/trimethoprim	>4/76	>4/76	>4/76
Piperacillin + Tazobactam	>16	>16	>16
Aztreonam	>32	>32	>32
Cefepime	>16	>16	>16
Eravacycline	>0.5	>0.5	>0.5
Imipenem	>8	>8	>8
Imipenem/relebactam	>8/4	>8/4	>8/4
Tigecycline	1	1	1
Tobramycin	>4	>4	>4
Cefiderocol	2	2	8

**Table 2:** Antibiotic resistance genes and mutations in iron uptake and transport genes identified in the three *Klebsiella pneumoniae* isolates. RS and BA1 strains showed a Cefiderocol MIC of 2 mg/L (categorized as susceptible), whereas BA2 showed a MIC of 8 mg/L (categorized as resistant).

Iron Uptake and Transport Genes		Antibiotic Resistance Genes			
Mutations	Gene	Genes	Gene Function	ST	Isolates
		aac(6′)-Ib4	Aminoglycoside resistance	ST307	RS, BA1
		aadA1			
		aph(3″)-Ib			
		aph(6)-Id			
		blaCTX-M-15	Beta-lactam resistance		
		blaOXA-1			
		blaSHV-12			
		blaTEM-206			
		blaVIM-1			
		emrD	Efflux		
		mph(A)	Macrolide		
		catB2	Phenicol		
		catB3			
		oqxB19	Phenicol/Quinolone		
		parC_S80I	Quinolone		
		gyrA_S83I			
		qnrB1			
		qnrS1			
		sul1	Sulfonamide		
		sul2			
		tet(A)	Tetracycline		
		dfrA14	Trimethoprim		
Ala231Val	*envZ*	aac(6′)-Ib4	Aminoglycoside resistance	ST307	BA2
		aadA1			
		aph(3″)-Ib			
		aph(6)-Id			
		blaCTX-M-15	Beta-lactam resistance		
		blaOXA-1			
		blaSHV-12			
		blaTEM-206			
		blaVIM-1			
		emrD	Efflux		
		mph(A)	Macrolide		
		catB2	Phenicol		
		catB3			
		oqxB19	Phenicol/Quinolone		
		parC_S80I	Quinolone		
		gyrA_S83I			
		qnrB1			
		qnrS1			
		sul1	Sulfonamide		
		sul2			
		tet(A)	Tetracycline		
		dfrA14	Trimethoprim		

## Data Availability

All the relevant research data of this study can be found in the article or in the Supplementary Materials. Raw genomic data of the bacterial isolates have been deposited on on-line repositories. Repository name and accession numbers can be found in the article (see Section 4.1).

## Supplementary Materials

The following supporting information can be downloaded at https://www.mdpi.com/article/10.3390/antibiotics14090893/s1. Figure S1: Growth curves of bacterial strains BA1 and BA2 measured as optical density (McFarland units) under iron-rich and iron-depleted conditions.

## Abbreviations

The following abbreviations are used in this manuscript:

CFD	cefiderocol
CRE	carbapenem-resistant *Enterobacterales*
MBL	metallo-β-lactamase
VAP	ventilator-associated pneumonias
TDM	therapeutic drug monitoring
MIC	minimum inhibitory concentration
