# Supplementary material for: Rapid Emergence of Cefiderocol Resistance Associated with Mutation of EnvZ Gene in a VIM-Producing ST307 Klebsiella pneumoniae Strain
Source: Antibiotics (Basel). 2025 Sep 4;14(9):893. doi: 10.3390/antibiotics14090893 (PMC12466864; doi:10.3390/antibiotics14090893)
Supplement: Supplementary file 1 [file antibiotics-14-00893-s001.zip › antibiotics-3811108-supplementary.pdf]

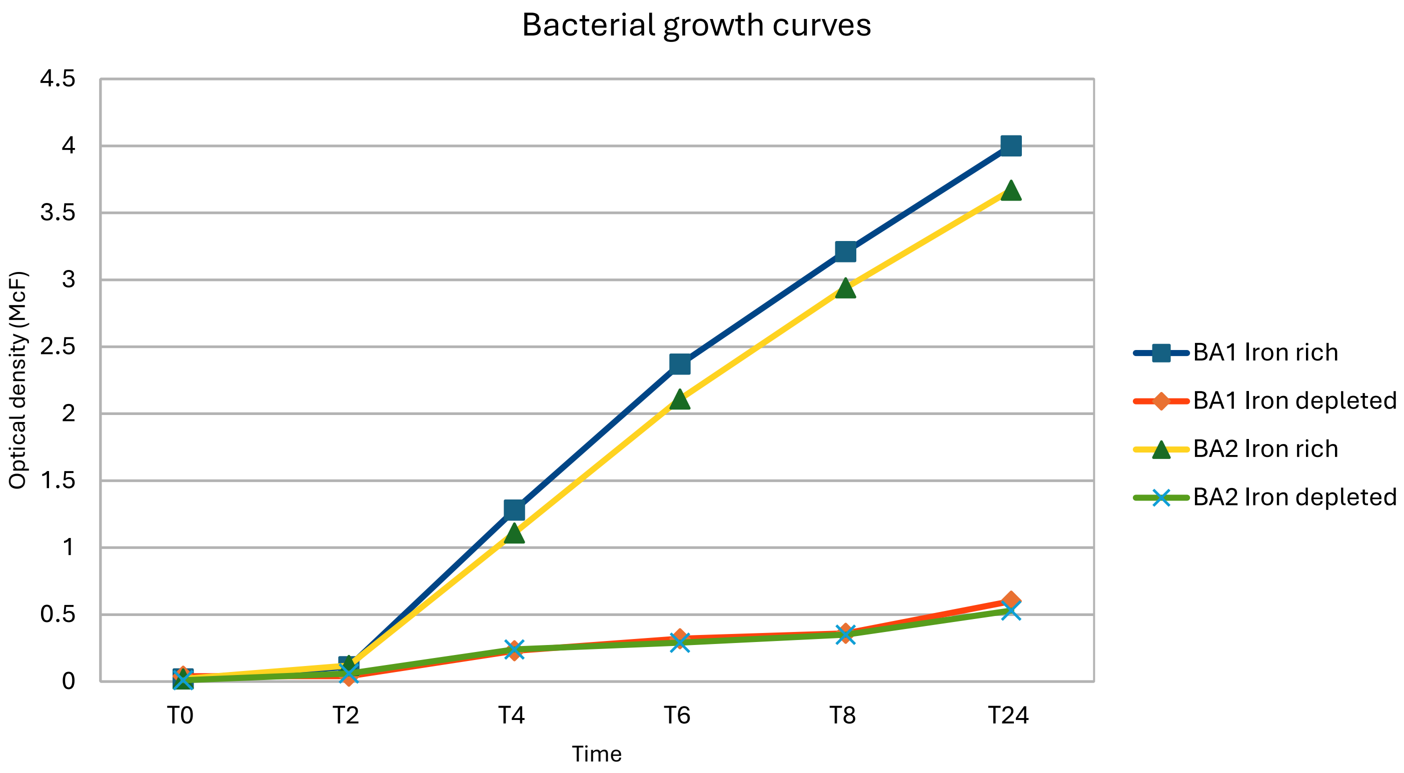

Supplementary Figure S1. Growth curves of bacterial strains BA1 and BA2 measured as optical density (McFarland units) under iron-rich and iron-depleted conditions. Data are shown at different time points (T0, T2, T4, T6, T8, T24), highlighting the impact of iron availability on bacterial growth dynamics.
